# Automatic contouring system for cervical cancer using convolutional neural networks

**DOI:** 10.1002/mp.14467

**Published:** 2020-10-09

**Authors:** Dong Joo Rhee, Anuja Jhingran, Bastien Rigaud, Tucker Netherton, Carlos E. Cardenas, Lifei Zhang, Sastry Vedam, Stephen Kry, Kristy K. Brock, William Shaw, Frederika O’Reilly, Jeannette Parkes, Hester Burger, Nazia Fakie, Chris Trauernicht, Hannah Simonds, Laurence E. Court

**Affiliations:** ^1^ MD Anderson UTHealth Graduate School Houston TX USA; ^2^ Department of Radiation Physics Division of Radiation Oncology The University of Texas MD Anderson Cancer Center Houston TX USA; ^3^ Department of Radiation Oncology The University of Texas MD Anderson Cancer Center Houston TX USA; ^4^ Department of Imaging Physics The University of Texas MD Anderson Cancer Center Houston TX USA; ^5^ Department of Medical Physics (G68) University of the Free State Bloemfontein South Africa; ^6^ Division of Radiation Oncology and Medical Physics University of Cape Town and Groote Schuur Hospital Cape Town South Africa; ^7^ Division of Medical Physics Stellenbosch University Tygerberg Academic Hospital Cape Town South Africa; ^8^ Division of Radiation Oncology Stellenbosch University Tygerberg Academic Hospital Cape Town South Africa

**Keywords:** auto‐contouring, cervical cancer, convolutional neural network, deep learning

## Abstract

**Purpose:**

To develop a tool for the automatic contouring of clinical treatment volumes (CTVs) and normal tissues for radiotherapy treatment planning in cervical cancer patients.

**Methods:**

An auto‐contouring tool based on convolutional neural networks (CNN) was developed to delineate three cervical CTVs and 11 normal structures (seven OARs, four bony structures) in cervical cancer treatment for use with the Radiation Planning Assistant, a web‐based automatic plan generation system. A total of 2254 retrospective clinical computed tomography (CT) scans from a single cancer center and 210 CT scans from a segmentation challenge were used to train and validate the CNN‐based auto‐contouring tool. The accuracy of the tool was evaluated by calculating the Sørensen‐dice similarity coefficient (DSC) and mean surface and Hausdorff distances between the automatically generated contours and physician‐drawn contours on 140 internal CT scans. A radiation oncologist scored the automatically generated contours on 30 external CT scans from three South African hospitals.

**Results:**

The average DSC, mean surface distance, and Hausdorff distance of our CNN‐based tool were 0.86/0.19 cm/2.02 cm for the primary CTV, 0.81/0.21 cm/2.09 cm for the nodal CTV, 0.76/0.27 cm/2.00 cm for the PAN CTV, 0.89/0.11 cm/1.07 cm for the bladder, 0.81/0.18 cm/1.66 cm for the rectum, 0.90/0.06 cm/0.65 cm for the spinal cord, 0.94/0.06 cm/0.60 cm for the left femur, 0.93/0.07 cm/0.66 cm for the right femur, 0.94/0.08 cm/0.76 cm for the left kidney, 0.95/0.07 cm/0.84 cm for the right kidney, 0.93/0.05 cm/1.06 cm for the pelvic bone, 0.91/0.07 cm/1.25 cm for the sacrum, 0.91/0.07 cm/0.53 cm for the L4 vertebral body, and 0.90/0.08 cm/0.68 cm for the L5 vertebral bodies. On average, 80% of the CTVs, 97% of the organ at risk, and 98% of the bony structure contours in the external test dataset were clinically acceptable based on physician review.

**Conclusions:**

Our CNN‐based auto‐contouring tool performed well on both internal and external datasets and had a high rate of clinical acceptability.

## 1 INTRODUCTION

Manual contouring of tumors and normal structures is a very labor‐intensive and time‐consuming part of the radiation treatment planning process.^1^, ^2^ “Wrong or inaccurate” contours drawn by physicians and dosimetrists constitute the highest and seventh‐highest risk factors for failure of photon/electron external beam radiation treatment, respectively.^3^ Most of these errors could be avoided if an accurate and reliable auto‐contouring tool were available. In the past, various algorithms have been evaluated for the development of auto‐contouring tools, with mixed success.^4^, ^5^, ^6^ With the advent of deep learning, more specifically, convolutional neural networks (CNNs), this movement has been accelerated as CNNs outperformed most of the other algorithms in various segmentation tasks.^7^ As a result, CNN‐based auto‐contouring systems for computed tomography (CT) images have been developed for various body sites, such as the head and neck,^8^, ^9^, ^10^, ^11^, ^12^ thoracic region,^13^, ^14^, ^15^, ^16^ abdomen,^17^, ^18^, ^19^ and pelvis.^20^, ^21^, ^22^, ^23^, ^24^, ^25^, ^26^, ^27^, ^28^, ^29^, ^30^, ^31^, ^32^

Although these approaches have generally been very successful, they are not yet accessible to cancer treatment centers where they would be most useful — those with limited resources that see a large number of cervical cancer patients, such as in South Africa and other low‐ and middle‐income countries (LMICs). In fact, cervical cancer is the second most common cancer in women in Africa,^33^, ^34^ and the most cost‐effective treatment that increases the survival rate of cervical cancer patients in LMICs is radiation treatment.^35^ To fill this gap, the Radiation Planning Assistant (RPA; rpa.mdanderson.org),^36^ a web‐based, fully automated radiotherapy contouring and planning generation system, is being developed to address the shortage of treatment planning staff and subsequently increase the survival rate for cancer patients in LMICs.

Although the potential of deep learning‐based auto‐contouring systems for pelvic structures has been explored in several previous studies, most of them were focused on prostate cancer,^21^, ^22^, ^23^, ^24^, ^31^, ^32^ and only a few papers have published results for the female pelvis.^25^, ^26^ In this study, we developed an auto‐contouring system that can contour the clinical treatment volumes (CTVs) and normal structures that are necessary for various cervical cancer radiation treatment planning techniques. The auto‐contouring system in this work will be implemented with RPA to automatically generate high‐quality radiation treatment for cervical cancer patients in LMICs.

To the best of our knowledge, this study is the first CT‐based auto‐contouring study that includes pelvic lymph node CTV (nodal CTV) and para‐aortic lymph node CTV (PAN CTV) for cervical cancer radiotherapy using deep learning. This addition is important, as these are the primary targets for radiotherapy treatments of cervical cancer and will facilitate fully automated treatment planning for cervical cancer.

## 2 MATERIALS AND METHODS

Our CNN‐based auto‐contouring tool was developed to generate contours for three CTVs and 11 normal structures in the female pelvis: primary CTV, nodal CTV, PAN CTV, bladder, rectum, spinal cord, left and right femurs, left and right kidneys, sacrum, pelvic bone, L4 vertebral body, and L5 vertebral body. These structures were categorized into three groups: bony structures, organs at risk (OARs), and CTVs. These are the structures required to automate 4‐field box, 3D conformal, IMRT, and VMAT plans for cervical cancer.^37^, ^38^, ^39^

**Fig. 1:**
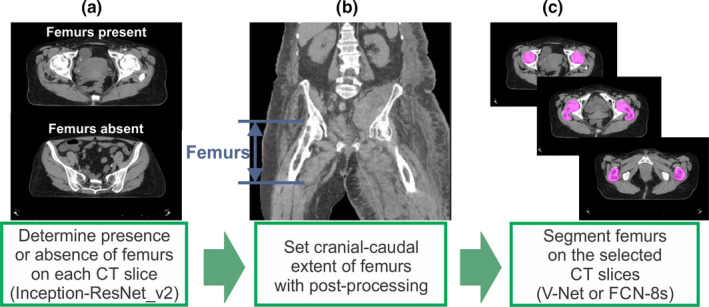
Application of the convolutional neural network‐based classification and segmentation models to a computed tomography (CT) scan. (a) The presence or absence of the organ of interest (in this case, femurs) was evaluated on each CT slice, (b) the cranial‐caudal extent of the organ of interest was determined with postprocessing, and (c) the slices that were classified to contain the organ of interest were used in the segmentation model to generate contours. [Color figure can be viewed at wileyonlinelibrary.com]

First, the Inception‐ResNet‐V2^40^ classification architecture was trained to identify the extent of the structure in the cranial‐caudal direction, as shown in Figs. 1(a) and 1(b). This approach was taken to address the GPU memory limitation issue as well as to improve the accuracy of the automatically generated contours by allowing the subsequent segmentation model to process a restricted field of view.^9^ Second, the segmentation models were applied to the CT slices that were classified to contain the organ of interest, as shown in Fig. 1(c). Both the classification and the segmentation models were trained independently for each structure.

### 2.A Training parameters

For the training and validation data, 2254 female pelvic CT scans from cancer patients who received radiation treatment from September 2004 to June 2018 at The University of Texas MD Anderson Cancer Center were used. Furthermore, 210 CT scans with kidney contours from the 2019 Kidney Tumor Segmentation Challenge (KiTS19) were added to our training data. The CT scans had pixel sizes in the transverse plane that ranged from 0.754 to 1.367 mm and slice thicknesses from 2.0 to 3.0 mm, except for 8 CT scans (3 were 5 mm, 3 were 4 mm, 1 was 1.5 mm, and 1 was 1.0 mm thick). All data were resampled to have the same voxel size of 1.17 mm × 1.17 mm × 2.5 mm. The CT numbers lower than −1000 HU and higher than 3000 HU were clipped and then linearly shifted to have a 0 to 4000 pixel intensity range.

An NVIDIA DGX Station with four V100 GPUs (16 GB RAM) was used to train our models. The loss function for the segmentation models was the Sørensen‐Dice similarity coefficient (DSC),^41^, ^42^ as this was our metric to determine the accuracy of the segmentation model. A weighted cross‐entropy was used as a loss function for the classification model to compensate for the data imbalance between the number of slices with and without the organ of interest. The weight was determined to be the ratio of the number of absences to the number of presences. The Adam optimizer^43^ was used as an optimization algorithm. The Adam optimizer’s parameters, beta1, beta2, and epsilon, were set to 0.9, 0.999, and 10^−8^, respectively.

To select the two‐dimensional (2D) and three‐dimensional (3D) CNN segmentation architectures, we did a preliminary study on the spinal cord for 2D and the left kidney for 3D. The vanilla DeepLabv3+^44^ and the FCN‐8s^45^ with additional batch normalization layers at the end of every convolutional layer were trained to segment the spinal cord in 2D. The mean ± standard deviation DSC were 0.87 ± 0.03 and 0.90 ± 0.02, for the vanilla DeepLabv3+ and the modified FCN8‐s, respectively, so the modified FCN‐8s was chosen for our model. Similarly, the 3D U‐Net^46^ and the 3D V‐Net^21^ segmentation architectures were trained to segment the left kidney on CT images resized to have a 256 × 256 × 60 dimension. We added batch normalization layers at the end of every convolutional layer for both architectures. The mean ± standard deviation DSC were 0.93 ± 0.04 and 0.93 ± 0.04, for the U‐Net and the V‐Net, respectively. As there was no significant difference between the two architectures, we chose the V‐Net, which has residual connections in each stage.

### 2.B Bony structures

The contours of the four bony structures (pelvic bone, sacrum, L4 vertebral body, and L5 vertebral body) were generated on 370 CT scans to train and validate the auto‐contouring model. The pelvic bone was defined to be the traditional pelvic bone without the sacrum, as the sacrum was contoured as a separate structure. All the bony structure contours were automatically generated with a multi‐atlas‐based auto‐contouring system (MACS)^4^, ^5^, ^47^ first, and the automatically generated contours were manually reviewed and revised if necessary.

V‐Net,^21^ a CNN‐based 3D segmentation architecture, was used to segment the four bony structures. The input image for the segmentation architecture was resized to N_slice_ × 256 × 256. A single segmentation model was used to contour the adjacent L4 and L5 vertebral bodies simultaneously. For data augmentation purposes, horizontal flip and rotation with random angles between −30° and 30° along the axial axis were applied for these structures.

### 2.C Clinical treatment volumes

#### 2.C.1 Primary clinical treatment volume

The primary CTV for cervical cancer patients is defined to include the uterus and the cervix. To train the model, 406 contours were either curated from clinical contours or manually generated from scratch by 4 physicians at MD Anderson Cancer Center.

**Fig. 2:**
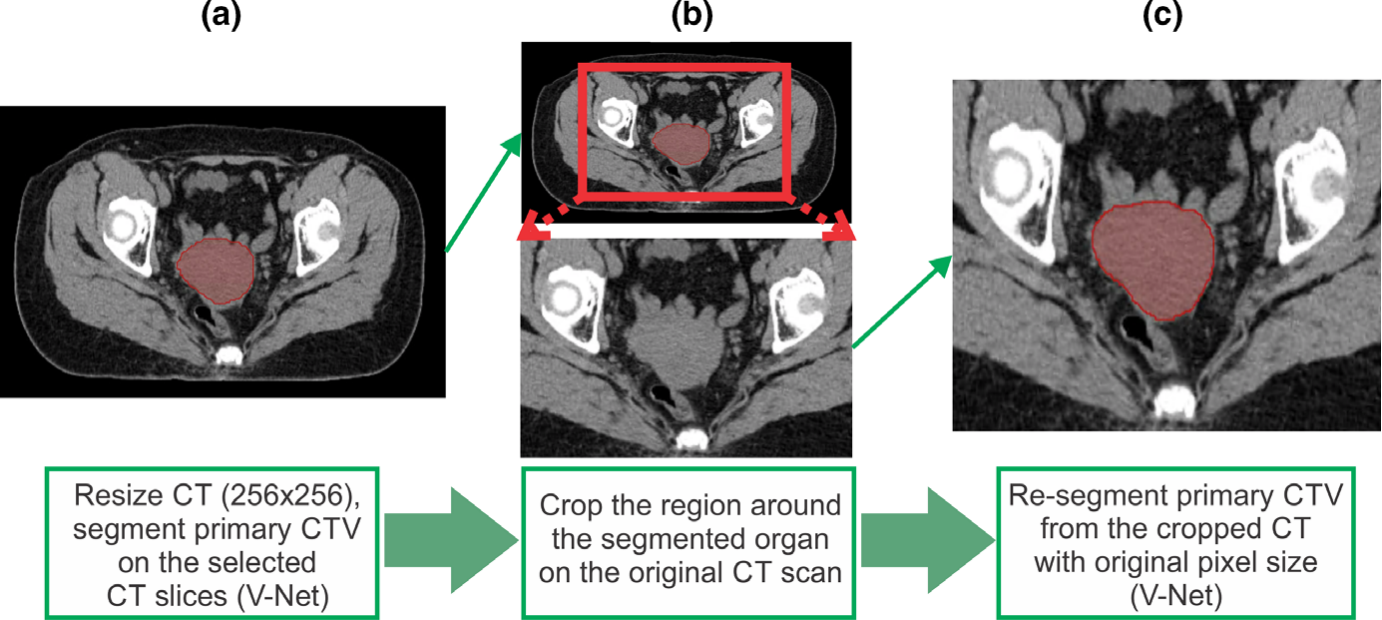
Segmentation using cropped three‐dimensional images for better accuracy. (a) Resize the computed tomography (CT) from 512 × 512 to 256 × 256 pixels and then segment the organ of interest and find the center of mass, (b) crop the region around the segmented organ on the original 512 × 512 CT scan, and (c) resegment the organ of interest on the cropped image. [Color figure can be viewed at wileyonlinelibrary.com]

V‐Net was used to segment the primary CTV. Although the classification model restricted the field of view of the input images, the GPU memory was not sufficient to train the full‐resolution CT images. To overcome this problem, we resized the input image to 256 × 256 pixels in the transverse plane, segmented the primary CTV, and estimated the center of mass of the primary CTV. Then, we cropped the box that fully enclosed the primary CTV and centered it on the center of mass of the prediction on the original CT scan with a 512 × 512 pixel image size. Finally, we applied the V‐Net segmentation model to the cropped 3D image, as shown in Fig. 2. This way, the final contour is predicted on the limited CT field of view with the original spatial resolution. This approach was inspired by the method proposed by Feng et al.^13^ and applied to the rest of the CTVs and OARs that were segmented with the 3D segmentation model.

Although the cropped images were supposed to be centered at the center of mass of the organ in the prediction, the center was randomly chosen while training the model in each epoch for the data augmentation purpose. Furthermore, the random rotation between −30° and 30° along the axial axis and the horizontal flip were also used for data augmentation. The same data augmentation techniques were applied to train the segmentation models for other CTVs and OARs.

#### 2.C.2 Pelvic lymph node clinical treatment volume

The nodal CTV covers the common iliac, external iliac, internal iliac, obturator, and presacral nodal regions as described in the GEC‐ESTRO II guideline^48^ for intermediate‐risk nodal CTV. To provide data for the training process, 250 nodal CTV contours were contoured by the same four physicians who contoured the primary CTV and later peer‐reviewed to ensure high accuracy and consistency. As the lymph nodes and vessels are small and have CT numbers similar to those of muscles, a 3D segmentation model can sometimes miss a small part of the lymph nodes. To prevent this, FCN‐8s,^45^ a 2D segmentation architecture, was also trained to auto‐contour the nodal CTVs. The CT slices that were predicted to contain the nodal CTV contours by the 3D segmentation model were given to the 2D segmentation model for slice by slice prediction. In prediction, the sum of the nodal CTV contours from the 2D and 3D models was used as a final contour.

The superior border of the intermediate nodal CTV was determined at one slice below the bifurcation of the common iliac artery. To locate the superior border more accurately, a segmentation model for the aorta near the bifurcation region was trained with 296 CT scans. The segmentation model was applied to a cropped region around the automatically generated L4 vertebral body contour to limit the field of view.

#### 2.C.3 Para‐aortic lymph node (PAN) clinical treatment volume

The PAN CTV covers the para‐aortic lymph nodes from the level of the renal veins to the aorta above the aortic bifurcation (i.e., one slice above the superior slice of the nodal CTV). In order to gather data sufficient for the PAN CTV segmentation model, we used 146 clinical contours, and all the contours were manually curated and revised if necessary. FCN‐8s was used to auto‐contour the PAN CTVs.

### 2.D Organs at risk

**Fig. 3:**
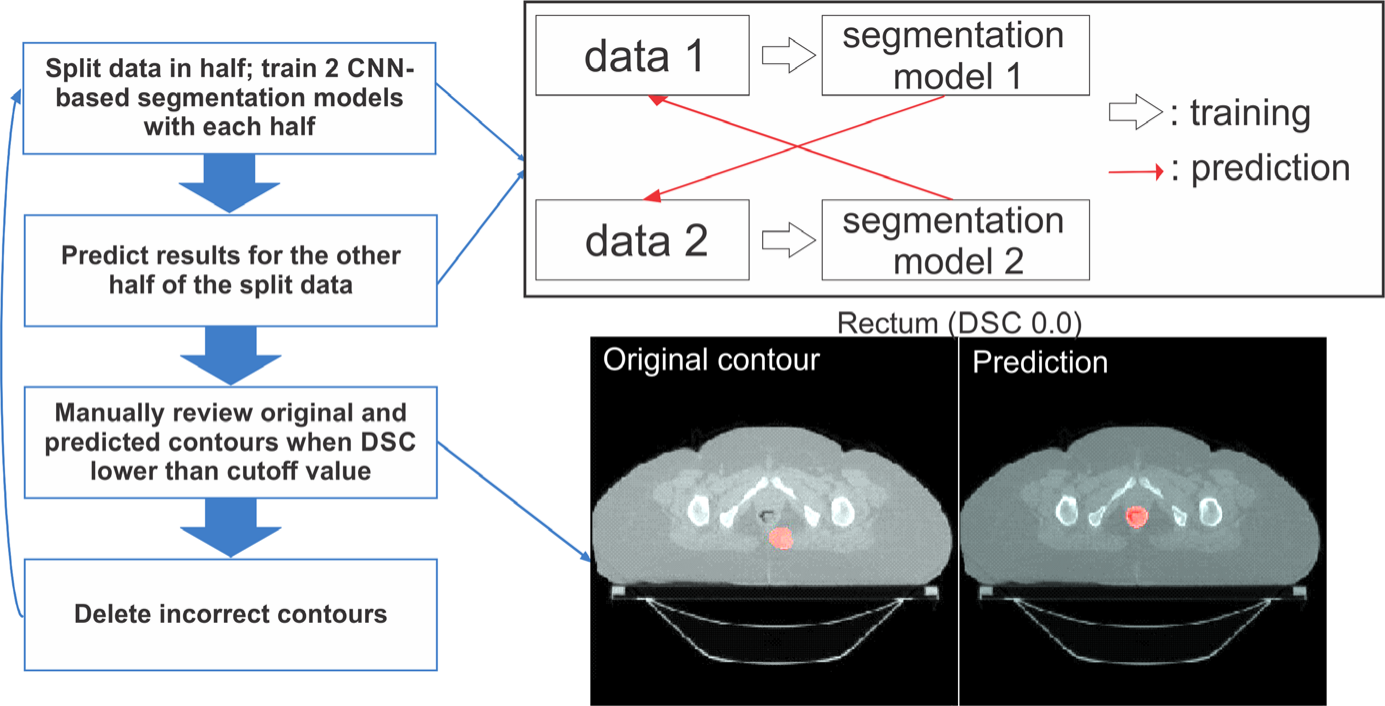
Flowchart of the semi‐automated data curation method to identify incorrect clinical contours. Data were randomly split into two groups, and two auto‐segmentation models were trained with each dataset. Then, each segmentation model was applied to the other group of data to create contours. If the Sørensen‐Dice similarity coefficient was lower than the threshold value, the original contour was manually reviewed and deleted if incorrect. [Color figure can be viewed at wileyonlinelibrary.com]

OARs for cervical cancer radiation treatment include the bladder, rectum, spinal cord, left and right femurs, and left and right kidneys. The training and validation data for the OARs were acquired from clinical contours of the 2254 CT scans. Contours for each structure were considered to maximize the amount of data, and thus, the number of available structures in a single patient’s data varied from 1 to 7. The total number of CT scans used for training and validation for each structure is shown in Table I. Of these scans, 80% were used for training, and 20% were used for validation. Since the classification and the segmentation models were trained independently for each structure to avoid the class imbalance problem,^49^ the imbalance in the number of training data for each structure did not influence the model accuracy. As the contours were collected solely on the basis of their labels, review of these contours was required to confirm their accuracy. Owing to the substantial number of contours, we proposed a semi‐automatic data curation method instead of manual review, as described in Fig. 3. First, “unreviewed” contours and the corresponding scans were divided in half. Two CNN‐based segmentation models, one for each half, were trained, and the contours were predicted on the other half of the dataset. If the DSC between the clinical contours and the predicted contours was lower than an arbitrarily determined threshold value (DSC = 0.7 for the rectum, 0.8 for the remaining OARs), the original contour was manually reviewed, and any incorrect clinical contours were removed from the training dataset. Once the entire set of training data was reviewed, we repeated the process with the “refined” dataset from the beginning three times.

**Table I:** The number of computed tomography (CT) scans used for training and validation for each structure.

Structure	Number of training and validation datasets
Primary CTV	406
Nodal CTV	250
PAN CTV	146
Bladder	1678
Rectum	1514
Spinal cord	655
Femurs (left, right)	962, 983
Kidneys (left, right)	907, 943
Pelvic bone	370
Sacrum L4/L5 vertebral bodies	370
	370

CTV: clinical treatment volume; PAN: para‐aortic lymph node.

The left and right kidney contours from the KiTS19 dataset^50^ were added to the training dataset. Abnormal kidneys with large tumors were excluded from the dataset, so 172 contours and 186 contours, respectively, for left and right kidneys were added from the total of 210 CT scans.

**Fig. 4:**
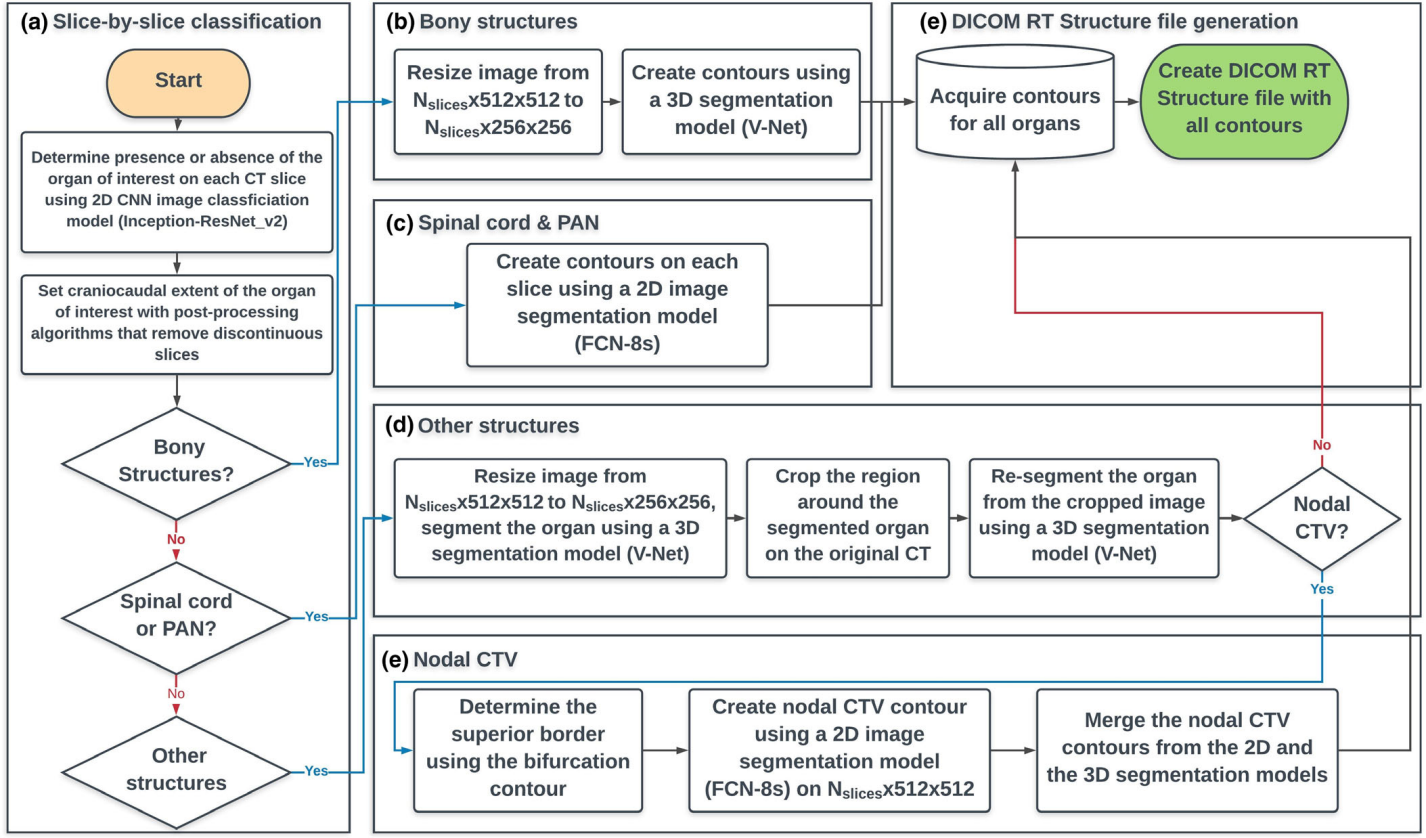
Overall flowchart of the developed auto‐contouring system for cervical cancer. (a) The slice by slice classification was conducted to identify computed tomography (CT) slices that contain a target structure, and the process is visually demonstrated in Fig. 1. (b) Bony structures were contoured as described in Section 2.B. (c) Spinal cord and PAN clinical treatment volume (CTV) were contoured with the 2D FCN‐8s segmentation architecture. (d) Other structures (the organs‐at‐risk and the primary and the nodal CTVs) were contoured as demonstrated in Fig. 2. (e) Extra steps were required for the nodal CTV contours as described in Section 2.C.2. [Color figure can be viewed at wileyonlinelibrary.com]

All the OARs, except for the spinal cord, used 3D V‐Net segmentation models and followed the steps described in Fig. 2. For the spinal cord segmentation, a 2D FCN‐8s model was used to generate the contour on each slice. The overall flowchart of the developed auto‐contouring system is demonstrated in Fig. 4.

### 2.E Test dataset

For quantitative analysis of the auto‐contouring system, CT scans and corresponding clinical contouring data from 140 female pelvic cancer patients who received radiation treatment at MD Anderson were used as the test dataset. All of the test CT scans were independent from the training and validation CT scans.

The contours of the CTVs were manually generated by physicians, and the contours of the bony structures and OARs were manually generated by medical physics researchers and reviewed by physicians. Some of the CT scans did not show all of the OARs, owing to the limited cranial‐caudal extent. As the superior border of the PAN CTV can be slightly different, depending on the location of pathological nodes and physician judgment, we modified the superior borders of the automatically generated PAN CTV on the basis of the manually generated ground truth contour. We did the same for the inferior borders of the rectum and the spinal cord for similar reasons. The accuracy of the model was measured by the DSC, mean surface distance (MSD), and Hausdorff distance (HD)^47^ between the automatically generated contours and the ground truth contours.

For qualitative analysis, contours were automatically generated using the auto‐contouring system (Fig. 4) for CT scans from 30 cervical cancer patients from three South African hospitals. This dataset was completely independent from the training dataset and the potential population target for the RPA system. The automatically generated contours were evaluated by an experienced radiation oncologist at MD Anderson and scored as needing no edits, minor edits, or major edits. For the contours scored as needing minor edits, revisions were preferred but not mandatory for the contours to be considered clinically acceptable.

## 3 RESULTS

### 3.A Model accuracy

The DSC, MSD, and HD between the automatically generated contours and the internal test dataset for 140 CT scans are given in Table II. Owing to the limited cranial‐caudal extent, only 132, 129, and 127 contours were evaluated for the PAN, L4/L5 vertebral bodies, and kidneys, respectively; two patients did not have nodal CTV and one patient did not have spinal cord contours. All the CTVs had mean DSC > 0.76, mean MSD < 0.27 cm, and mean HD < 2.09 cm. All the normal structures had mean DSC > 0.81, mean MSD < 0.18 cm, and mean HD < 1.66 cm. All the bony structures had mean DSC > 0.90, mean MSD < 0.08 cm, and mean HD < 1.25 cm.

**Table II:** Sørensen‐Dice similarity coefficients (in percentage), mean surface distance (in cm), and Hausdorff distance (in cm) between our CNN‐based model and clinical contours from 140 internal test CT scans.

Structure	DSC (mean ± SD)	MSD (mean ± SD)	Hausdorff Distance (mean ± SD)
Primary CTV	0.86 ± 0.08	0.19 ± 0.12	2.02 ± 1.17
Nodal CTV	0.81 ± 0.03	0.21 ± 0.05	2.09 ± 0.56
PAN CTV	0.76 ± 0.09	0.27 ± 0.16	2.00 ± 1.00
Bladder	0.89 ± 0.09	0.11 ± 0.13	1.07 ± 0.89
Rectum	0.81 ± 0.09	0.18 ± 0.14	1.66 ± 1.17
Spinal cord	0.90 ± 0.02	0.06 ± 0.01	0.65 ± 0.18
Femur, left	0.94 ± 0.03	0.06 ± 0.03	0.60 ± 0.41
Femur, right	0.93 ± 0.04	0.07 ± 0.04	0.66 ± 0.43
Kidney, left	0.94 ± 0.02	0.08 ± 0.03	0.76 ± 0.28
Kidney, right	0.95 ± 0.02	0.07 ± 0.03	0.84 ± 0.37
Pelvic bone	0.93 ± 0.02	0.05 ± 0.02	1.06 ± 0.53
Sacrum	0.91 ± 0.02	0.07 ± 0.05	1.25 ± 1.12
L4 vertebral body	0.91 ± 0.15	0.07 ± 0.15	0.53 ± 0.36
L5 vertebral body	0.90 ± 0.15	0.08 ± 0.23	0.68 ± 0.81

CNN: convolutional neural network; CT: computed tomography; DSC: Sørensen‐Dice similarity coefficient; MSD: mean surface distance; SD: standard deviation.

**Fig. 5:**
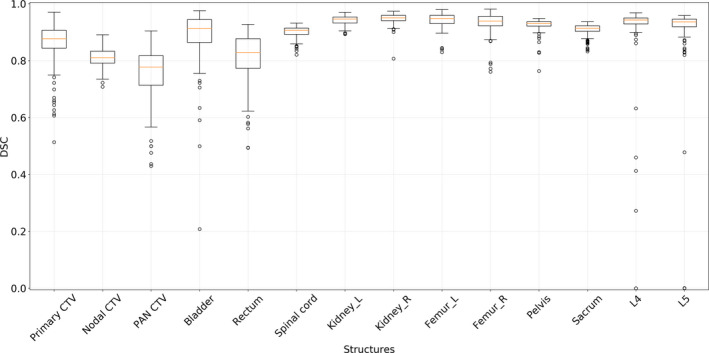
The distributions of Sørensen‐Dice similarity coefficients between the ground truth and the automatically generated contours of 14 structures. [Color figure can be viewed at wileyonlinelibrary.com]

**Fig. 6:**
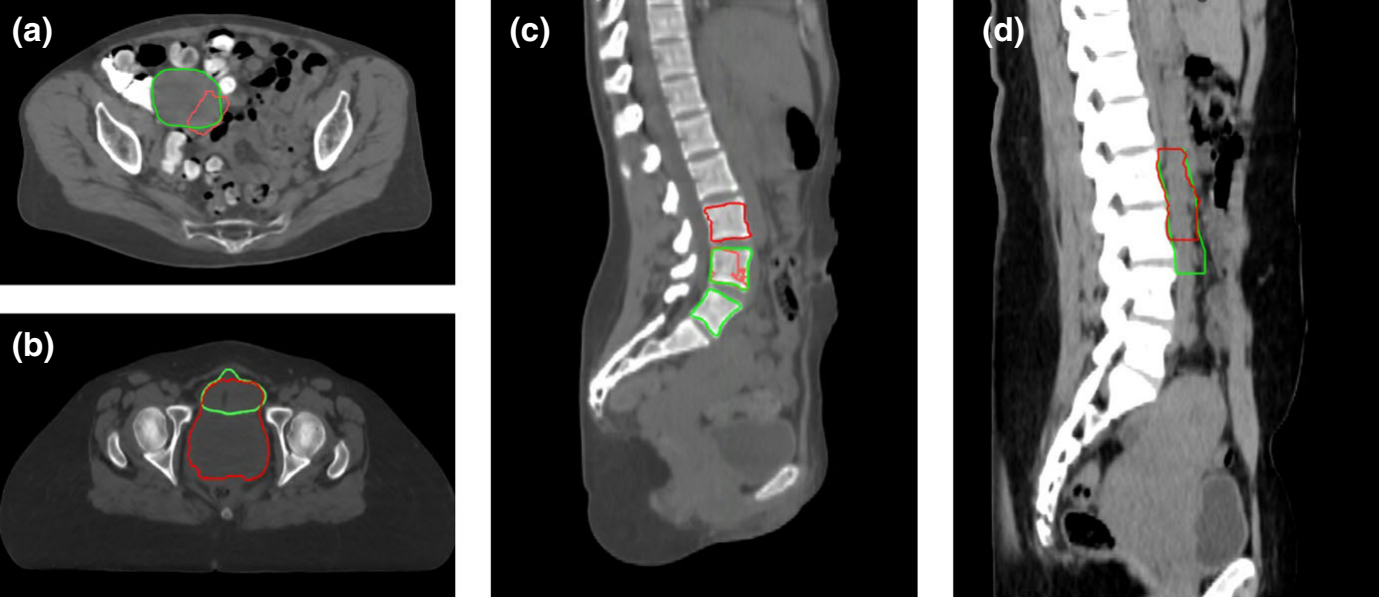
The outlier contours from the internal test dataset. The ground truth contours (green) and outliers (red) are given for (a) primary clinical treatment volume (CTV) (Sørensen‐Dice similarity coefficient [DSC] = 0.43), (b) bladder (DSC = 0.21), (c) L4 and L5 vertebral bodies (DSC = 0.0 each), and (d) PAN CTV (DSC = 0.43). [Color figure can be viewed at wileyonlinelibrary.com]

The overall boxplots of DSC for each structure are given in Fig. 5. Although most of the automatically generated contours had DSC distribution within a certain range, low DSC outliers existed in the box plots, and some of these contours are shown in Fig. 6. The failures in generating accurate contours often occurred when the CTVs and OARs were located near high‐density material in the bowel, as shown in Fig. 6(a). Contouring of the bladder occasionally failed when the border between the bladder and the uterus was vague, as shown in Fig. 6(b). Contouring of L4 and L5 vertebral bodies sometimes failed when the segmentation model predicted L3 to be L4 and L4 to be L5, as shown in Fig. 6(c). The automatically generated PAN CTV contours had low DSC values when the interface between the nodal CTV and the PAN CTV was incorrectly determined, as shown in Fig. 6(d).

### 3.B Physician review

**Fig. 7:**
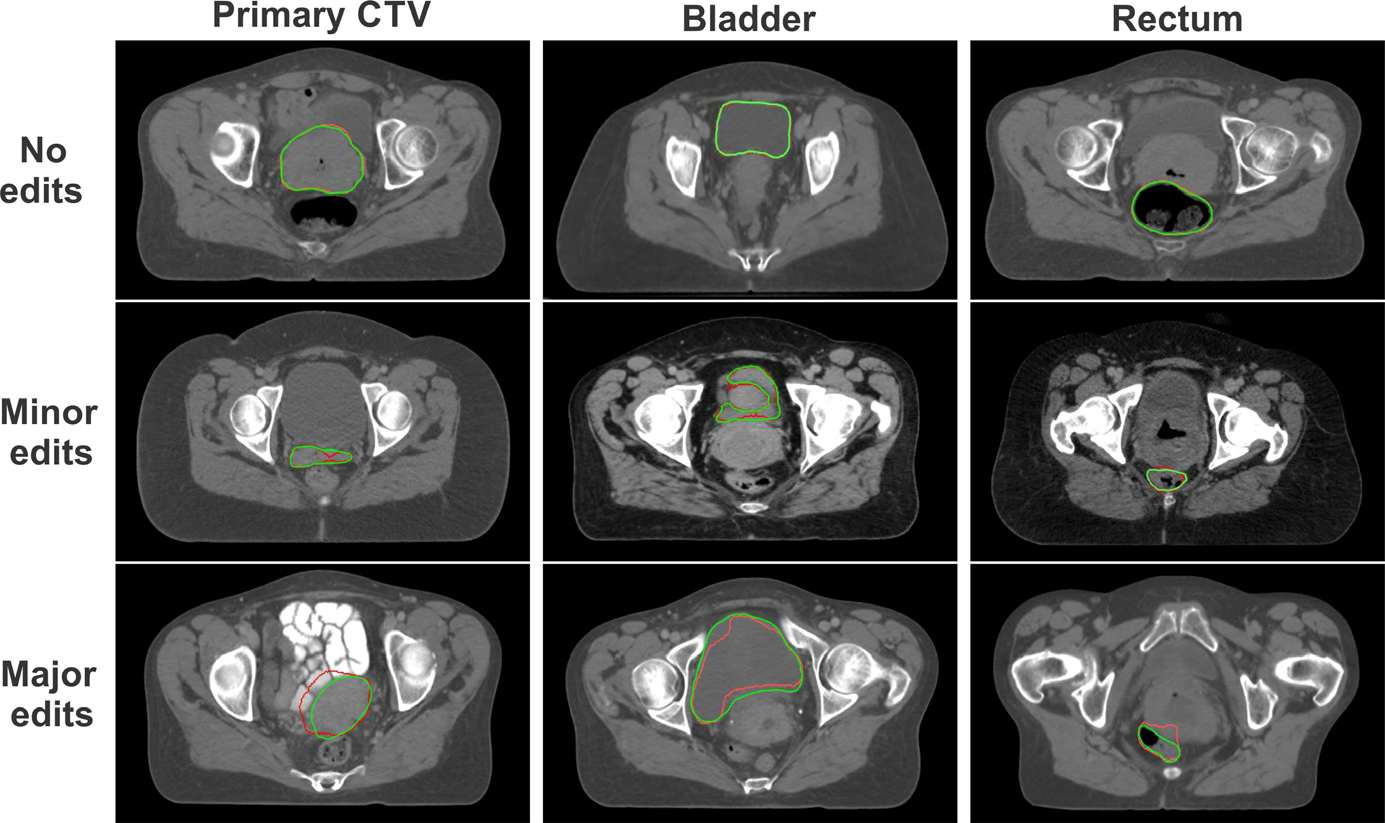
Examples of automatically generated contours (red) vs ground truth (green) from physician’s manual review of contours for the primary clinical treatment volume, bladder, and rectum. [Color figure can be viewed at wileyonlinelibrary.com]

Physician scoring of the automatically generated contours on the 30 external CT scans is shown in Table III. Owing to the limited cranial‐caudal extent, 28 contours were evaluated for the left and right kidneys. For the primary, nodal, and PAN CTVs, 83%, 70%, and 87% of the contours were clinically acceptable, respectively. For the bladder, rectum, and right kidney, 90%, 93%, and 96% were clinically acceptable, respectively, and the other OARs were 100% clinically acceptable. For the bony structures, 93% and 97% of the L4 and L5 vertebral bodies were clinically acceptable, respectively, and the pelvic bone and sacrum were 100% clinically acceptable. Some of the minor edits and major edits are demonstrated in Fig. 7.

**Table III:** Qualitative scores of the automatically generated contours on 30 external CT scans.

Structure	No edits (%)	Minor edits (%)	Major edits (%)
Primary CTV	8 (27%)	17 (57%)	5 (17%)
Nodal CTV	9 (30%)	12 (40%)	9 (30%)
PAN CTV	18 (60%)	8 (27%)	4 (13%)
Bladder	22 (73%)	5 (17%)	3 (10%)
Rectum	20 (67%)	8 (27%)	2 (7%)
Spinal cord	30 (100%)	0 (0%)	0 (0%)
Femur, left	27 (90%)	3 (10%)	0 (0%)
Femur, right	27 (90%)	3 (10%)	0 (0%)
Kidney, left	23 (82%)	5 (18%)	0 (0%)
Kidney, right	23 (82%)	4 (14%)	1 (4%)
Pelvic bone	24 (80%)	6 (20%)	0 (0%)
Sacrum	23 (77%)	7 (23%)	0 (0%)
L4 vertebral body	27 (90%)	1 (3%)	2 (7%)
L5 vertebral body	26 (87%)	3 (10%)	1 (3%)

CT: computed tomography; CTV: clinical treatment volume.

## 4 DISCUSSION

We have developed a CNN‐based auto‐contouring tool for three CTVs and 11 normal structures in cervical cancer CTs that can be used for fully automated radiation treatment planning. The number of training, validation, and test CT scans we used to train and evaluate this model is the largest to date among deep learning‐based female pelvis auto‐contouring studies.^25^, ^26^ We successfully acquired this high volume of data by using a semi‐automatic data curation method. Also, to the best of our knowledge, we are the first to auto‐contour nodal and PAN CTVs in the female pelvic region using deep learning. We have demonstrated that our CNN‐based auto‐contouring system can accurately generate clinically acceptable contours for both CTVs and normal structures in multiple patient cohorts.

### 4.A Quantitative results

For the bony structures, 3.5% (5/140 from the quantitative analysis) of the L4 and L5 vertebral body contours were not clinically acceptable (i.e. outliers in the boxplot in Fig. 5). Similarly, 6.7% (2/30 from the qualitative analysis) of the L4 and L5 vertebral body contours were not clinically acceptable (Table III). Therefore, the overall failure rate for the bony structures was about 4%. This is a noticeable improvement compared to a previous study where the failure rate for the automatically generated contours in a multi‐atlas‐based auto‐contouring system was about 10%.^39^

The performances of deep learning‐based auto‐contouring systems for OARs in the female pelvis from other published literature are presented in Table IV. As there is only one published paper on a deep learning‐based auto‐contouring system for cervical cancer, we have also included the state‐of‐the‐art auto‐contouring models for rectal and prostate cancers. Overall, the performance of our system is equivalent to or better than the auto‐contouring system for cervical cancer developed by Liu et al.^26^ for most of the structures.

**Table IV:** Summary of CNN‐based auto‐contouring results for normal structures in pelvic CTs from other groups.

Author	Sites	# test CTs	Structures	DSC results
Men et al. (2017)^29^	Rectal	60	Bladder	0.93
			Colon	0.62
			Intestine	0.65
			Femur_L	0.92
			Femur_R	0.92
			Rectal CTV	0.88
Kazemifar et al. (2018)^23^	Prostate	~26	Prostate	0.88
		(30% of 85)	Bladder	0.95
			Rectum	0.92
Balagopal et al. (2018)^24^	Prostate	~27	Prostate	0.90
		(Leave‐one‐out	Bladder	0.95
		cross‐validation,	Rectum	0.84
		20% of 135)	Femur_L	0.96
			Femur_R	0.95
Liu et al. (2020)^26^	Cervix	14	Bladder	0.92
			Bone marrow	0.85
			Rectum	0.79
			Small intestine	0.83
			Spinal cord	0.83
			Femur_L	0.91
			Femur_R	0.90
Our method	Cervix	140	Primary CTV (UteroCervix)	0.85
			Bladder (cervical cancer)	0.89
			Bladder (prostate cancer)	0.95
			Rectum	0.80
			Spinal cord	0.90
			Pelvic bone	0.93
			Sacrum	0.91
			Femur_L	0.94
			Femur_R	0.93

CNN: convolutional neural network; CT: computed tomography; DSC: Sørensen‐Dice similarity coefficient; CTV: clinical treatment volume.

Our quantitative test CT scans were randomly chosen from CTs of any female patient with an intact uterus, so the shape and volume of the bladder in the CT scans varied significantly. When we retrospectively tested our bladder segmentation model on 510 prostate patients with full bladders, the mean DSC was much improved at 0.95 ± 0.04. Compared with the state‐of‐the‐art rectal and prostate models, our model performed at least as well in all structures except for the rectum. However, the direct comparison of auto‐contouring models for different sites is not straightforward because the homogeneity of the structures in the test CT scans substantially changes the DSC, as shown in the accuracy of our two bladder models.

### 4.B Failure cases from physician’s review

The overall clinical acceptance rates were higher than 70% for the CTVs and 90% for the OARs and bony structures. When high‐density materials were located in the bowel, the auto‐contouring system had a higher chance of creating inaccurate contours of the CTVs or OARs near the region, as shown in Figs. 5 and 6. These high‐density materials were fecal matter resulting from a high‐carb diet with minimal protein, fat, and fibers, which likely causes compacted slow‐moving feces. This diet is more common in South Africa, the patient population for the external test dataset, than in the U.S, the patient population for the training and internal test datasets. As we acquire more CT data from such patients through the RPA system, we will be able to upgrade the auto‐contouring system to achieve more robust results in these patients.

**Fig. 8:**
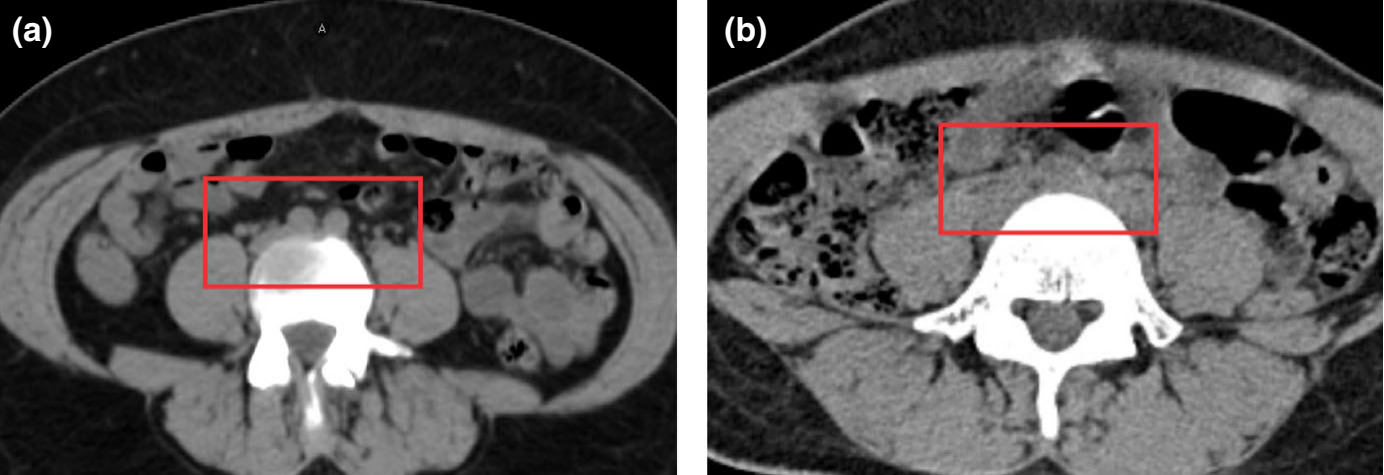
The aortic bifurcation is clearly defined in the red box in (a), whereas the aortic bifurcation is barely identifiable in the red box in (b). The adjustment of the window level did not improve the visual inspection. [Color figure can be viewed at wileyonlinelibrary.com]

For the nodal CTVs, 9/30 were scored as needing major edits; one was due to high‐density fecal matter in the bowel and three were due to failure to detect the superior border. The three cases did not have clear borders for vessels, as shown in Fig. 8(b), and therefore, the bifurcation segmentation model did not perform appropriately. All three patients seemed to be underweight, based on their CT scans, so we believe that the poor contrast resolution was due to incorrect use of image acquisition parameters or the lack of fat in between the vessels. We need to further investigate our auto‐contouring system in underweight patients and may need to adjust the CT acquisition parameters for these patients in the future.

We trained our model using a consistent, well‐curated dataset from a single hospital and the publicly available kidney contours. Final physician review used images from three other hospitals, with different patient populations from the training dataset. Thus, the review results gave us some confidence in the ability of our model to successfully contour patients from a different patient population, as well as various CT scanners and imaging protocols. In this study, we did not examine the impact of inter‐user variations on the physician assessment of these contours. Based on our experience with other sites,^51^ it is likely that an increased fraction of patients will be considered ‘minor edits’ instead of ‘no edits’ as we deploy the auto‐contouring system to more hospitals. We will further assess and quantify the inter‐user variability as we begin to deploy this system clinically.

## 5 CONCLUSION

We have demonstrated through both quantitative and qualitative studies that a CNN‐based, auto‐contouring tool can achieve clinically acceptable contours for most of the CTVs and normal structures in cervical cancer patients. We will implement our auto‐contouring system to the Radiation Planning Assistant, accelerating the radiation treatment planning process in hospitals in low‐ and middle‐income countries.

## CONFLICTS OF INTEREST

This work was partially funded by the National Cancer Institute and Varian Medical Systems.

## References

[1] Vorwerk H , Zink K , Schiller R , et al. Protection of quality and innovation in radiation oncology: the prospective multicenter trial the German Society of Radiation Oncology (DEGRO‐QUIRO study). Strahlentherapie und Onkol. 2014;190:433–443. 10.1007/s00066-014-0634-0 24595416

[2] Andrianarison VA , Laouiti M , Fargier‐Bochaton O , et al. Contouring workload in adjuvant breast cancer radiotherapy. Cancer Radiother. 2018;22:747–753. 30322819 10.1016/j.canrad.2018.01.008

[3] Ford E , Conroy L , Dong L , et al. Strategies for effective physics plan and chart review in radiation therapy: report of AAPM Task Group 275. Med Phys. 2020;47:e236–e272. 31967655 10.1002/mp.14030 PMC9012523

[4] Yang J , Haas B , Fang R , et al. Atlas ranking and selection for automatic segmentation of the esophagus from CT scans. Phys Med Biol. 2017;62:9140–9158. 29049027 10.1088/1361-6560/aa94ba PMC6167015

[5] Yang J , Zhang Y , Zhang L , Dong L . Automatic segmentation of parotids from CT scans using multiple atlases Medical Image Analysis for the Clinic: A Grand Challenge. 2010:223–230.

[6] Macomber MW , Phillips M , Tarapov I , et al. Autosegmentation of prostate anatomy for radiation treatment planning using deep decision forests of radiomic features. Phys Med Biol. 2018;63:235002. 30465543 10.1088/1361-6560/aaeaa4

[7] Cardenas CE , Yang J , Anderson BM , Court LE , Brock KB . Advances in auto‐segmentation. Semin Radiat Oncol. 2019;29:185–197. 31027636 10.1016/j.semradonc.2019.02.001

[8] Nikolov S , Blackwell S , Mendes R , et al. Deep learning to achieve clinically applicable segmentation of head and neck anatomy for radiotherapy. Prepr ArXiv; 2018:1–31. 10.2196/26151 PMC8314151 34255661

[9] Rhee DJ , Cardenas CE , Elhalawani H , et al. Automatic detection of contouring errors using convolutional neural networks. Med Phys. 2019;46:5089–5097. 10.1002/mp.13814 PMC6842055 31505046

[10] Zhu W . AnatomyNet Deep 3D Squeeze‐and‐excitation U‐Nets for fast and fully automated whole‐volume anatomical segmentation; 2018:1–14.

[11] Cardenas CE , McCarroll RE , Court LE , et al. Deep learning algorithm for auto‐delineation of high‐risk oropharyngeal clinical target volumes with built‐in dice similarity coefficient parameter optimization function. Int J Radiat Oncol. 2018;101:468–478. 10.1016/j.ijrobp.2018.01.114 PMC7473446 29559291

[12] Ibragimov B , Xing L . Segmentation of organs‐at‐risks in head and neck CT images using convolutional neural networks. Med Phys. 2017;44:547–557. 28205307 10.1002/mp.12045 PMC5383420

[13] Feng X , Qing K , Tustison NJ , Meyer CH , Chen Q . Deep convolutional neural network for segmentation of thoracic organs‐at‐risk using cropped 3D images. Med Phys. 2019;46:2169–2180. 30830685 10.1002/mp.13466

[14] Wang S , Zhou M , Gevaert O , et al.A multi‐view deep convolutional neural networks for lung nodule segmentation. In: 2017 39th Annual International Conference of the IEEE Engineering in Medicine and Biology Society (EMBC); 2017:1752–1755. 10.1109/EMBC.2017.8037182 29060226

[15] McIntosh C , Svistoun I , Purdie TG . Groupwise conditional random forests for automatic shape classification and contour quality assessment in radiotherapy planning. IEEE Trans Med Imaging. 2013;32:1043–1057. 23475352 10.1109/TMI.2013.2251421

[16] Lustberg T , Van SJ , Gooding M , et al. Clinical evaluation of atlas and deep learning based automatic contouring for lung cancer. Radiother Oncol. 2018;126:312–317. 29208513 10.1016/j.radonc.2017.11.012

[17] Roth HR , Oda H , Zhou X , et al. An application of cascaded 3D fully convolutional networks for medical image segmentation. Comput Med Imaging Graph. 2018;66:90–99. 29573583 10.1016/j.compmedimag.2018.03.001

[18] Hu P , Wu F , Peng J , Bao Y , Chen F , Kong D . Automatic abdominal multi‐organ segmentation using deep convolutional neural network and time‐implicit level sets. Int J Comput Assist Radiol Surg. 2017;12:399–411. 27885540 10.1007/s11548-016-1501-5

[19] Zhou X , Takayama R , Wang S , Hara T , Fujita H . Deep learning of the sectional appearances of 3D CT images for anatomical structure segmentation based on an FCN voting method. Med Phys. 2017;44:5221–5233. 28730602 10.1002/mp.12480

[20] Yang X , Yu L , Wu L , et al.Fine‐grained Recurrent Neural Networks for Automatic Prostate Segmentation in Ultrasound Images. December 2016 http://arxiv.org/abs/1612.01655. Accessed May 2, 2018.

[21] Milletari F , Navab N , Ahmadi SA .V‐Net: Fully convolutional neural networks for volumetric medical image segmentation. Proc ‐ 2016 4th Int Conf 3D Vision, 3DV 2016; 2016:565–571. 10.1109/3DV.2016.79

[22] Liu C , Gardner SJ , Wen N , et al. Automatic segmentation of the prostate on CT images using deep neural networks (DNN). Int J Radiat Oncol Biol Phys. 2019;104:924–932. 30890447 10.1016/j.ijrobp.2019.03.017

[23] Kazemifar S , Balagopal A , Nguyen D , et al. Segmentation of the prostate and organs at risk in male pelvic CT images using deep learning. Biomed Phys Eng Express. 2018;4:55003.

[24] Balagopal A , Kazemifar S , Nguyen D , et al. Fully automated organ segmentation in male pelvic CT images. Phys Med Biol. 2018;63:245015. 30523973 10.1088/1361-6560/aaf11c

[25] Breto AL , Zavala‐Romero O , Asher D , et al. A deep learning pipeline for per‐fraction automatic segmentation of GTV and OAR in cervical cancer. Int J Radiat Oncol Biol Phys. 2019;105:S202.

[26] Liu Z , Liu X , Xiao B , et al. Segmentation of organs‐at‐risk in cervical cancer CT images with a convolutional neural network. Phys Medica. 2020;69:184–191. 10.1016/j.ejmp.2019.12.008 31918371

[27] Cha KH , Hadjiiski LM , Samala RK , et al. Bladder cancer segmentation in CT for treatment response assessment: application of deep‐learning convolution neural network‐A Pilot study. Tomogr (Ann Arbor, Mich). 2016;2:421–429. 10.18383/j.tom.2016.00184 PMC5241049 28105470

[28] Cheng R , Roth HR , Lay N , et al. Automatic magnetic resonance prostate segmentation by deep learning with holistically nested networks. J Med imaging (Bellingham, Wash). 2017;4:41302. 10.1117/1.JMI.4.4.041302 PMC5565676 28840173

[29] Men K , Dai J , Li Y . Automatic segmentation of the clinical target volume and organs at risk in the planning CT for rectal cancer using deep dilated convolutional neural networks. Med Phys. 2017;44:6377–6389. 28963779 10.1002/mp.12602

[30] Trebeschi S , van Griethuysen JJM , Lambregts DMJ , et al. Deep learning for fully‐automated localization and segmentation of rectal cancer on multiparametric MR. Sci Rep. 2017;7:5301. 28706185 10.1038/s41598-017-05728-9 PMC5509680

[31] Karimi D , Samei G , Kesch C , Nir G , Salcudean SE . Prostate segmentation in MRI using a convolutional neural network architecture and training strategy based on statistical shape models. Int J Comput Assist Radiol Surg. 2018;13:1211–1219. 29766373 10.1007/s11548-018-1785-8

[32] To MNN , Vu DQ , Turkbey B , Choyke PL , Kwak JT . Deep dense multi‐path neural network for prostate segmentation in magnetic resonance imaging. Int J Comput Assist Radiol Surg. 2018;13:1687–1696. 30088208 10.1007/s11548-018-1841-4 PMC6177294

[33] Ferlay J , Soerjomataram I , Dikshit R , et al. Cancer incidence and mortality worldwide: sources, methods and major patterns in GLOBOCAN 2012. Int J cancer. 2015;136:E359–E386. 25220842 10.1002/ijc.29210

[34] Miller KD , Siegel RL , Lin CC , et al. Cancer treatment and survivorship statistics, 2016. CA Cancer J Clin. 2016;66:271–289. 27253694 10.3322/caac.21349

[35] Debas HT, Donkor P, Gawande A, et al. Essential Surgery: Disease Control Priorities. 3rd edn, Vol. 1 Washington (DC): The International Bank for Reconstruction and Development / The World Bank; April 2, 2015. 26740991

[36] Court LE , Kisling K , McCarroll R , et al. Radiation planning assistant ‐ a streamlined, fully automated radiotherapy treatment planning system. J Vis Exp. 2018:e57411 10.3791/57411 PMC5933447 29708544

[37] Chuang LT , Temin S , Camacho R , et al. Management and care of women with invasive cervical cancer: American Society of Clinical Oncology resource‐stratified clinical practice guideline. J Glob Oncol. 2016;2:311–340. 28717717 10.1200/JGO.2016.003954 PMC5493265

[38] International Atomic Energy Agency . Management of Cervical Cancer: Strategies for Limited‐resource Centres ‐ A Guide for Radiation Oncologists. Human Health Reports No. 6. Vienna: IAEA; Published 2013. https://www.iaea.org/publications/8738/management‐of‐cervical‐cancer‐strategies‐for‐limited‐resource‐centres‐a‐guide‐for‐radiation‐oncologists

[39] Kisling K , Zhang L , Simonds H , et al. Fully automatic treatment planning for external‐beam radiation therapy of locally advanced cervical cancer: a tool for low‐resource clinics. J Glob Oncol. 2019;5:1–9. 10.1200/JGO.18.00107 PMC6426517 30629457

[40] Szegedy C , Ioffe S , Vanhoucke V . Inception‐v4, Inception‐ResNet and the Impact of Residual Connections on Learning. CoRR. 2016;abs/1602.0.

[41] Smistad E , Østvik A , Haugen BO , L⊘vstakken L . 2D left ventricle segmentation using deep learning. In: 2017 IEEE International Ultrasonics Symposium (IUS); 2017:1–4. 10.1109/ULTSYM.2017.8092573

[42] Sørensen TJ . A Method of Establishing Groups of Equal Amplitude in Plant Sociology Based on Similarity of Species Content and Its Application to Analyses of the Vegetation on Danish Commons. København: I kommission hos E. Munksgaard; 1948.

[43] Kingma DP , Adam BJ . A Method for Stochastic Optimization. CoRR; 2014: abs/1412.6.

[44] Chen L‐C , Zhu Y , Papandreou G , Schroff F , Adam H . Encoder‐Decoder with Atrous Separable Convolution for Semantic Image Segmentation. February 2018. http://arxiv.org/abs/1802.02611. Accessed October 21, 2019.

[45] Shelhamer E , Long J , Darrell T . Fully convolutional networks for semantic segmentation. IEEE Trans Pattern Anal Mach Intell. 2017;39:640–651. 27244717 10.1109/TPAMI.2016.2572683

[46] Çiçek Ö , Abdulkadir A , Lienkamp SS , Brox T , Ronneberger O .3D U‐Net: Learning Dense Volumetric Segmentation from Sparse Annotation; June 2016 http://arxiv.org/abs/1606.06650. Accessed October 21, 2019.

[47] Yang J , Amini A , Williamson R , et al. Automatic contouring of brachial plexus using a multi‐atlas approach for lung cancer radiation therapy. Pract Radiat Oncol. 2013;3:e139–e147. 24674411 10.1016/j.prro.2013.01.002

[48] Pötter R , Haie‐Meder C , Van LE , et al. Recommendations from gynaecological (GYN) GEC ESTRO working group (II): concepts and terms in 3D image‐based treatment planning in cervix cancer brachytherapy—3D dose volume parameters and aspects of 3D image‐based anatomy, radiation physics, radiobiology. Radiother Oncol. 2006;78:67–77. 16403584 10.1016/j.radonc.2005.11.014

[49] Buda M , Maki A , Mazurowski M . A systematic study of the class imbalance problem in convolutional neural networks. Neur Netw. 2017;106:249–259. 10.1016/j.neunet.2018.07.011 30092410

[50] Heller N , Sathianathen N , Kalapara A , et al. The kits19 challenge data: 300 kidney tumor cases with clinical context, ct semantic segmentations, and surgical outcomes. arXiv Prepr arXiv190400445; 2019.

[51] McCarroll RE , Beadle BM , Balter PA , et al. Retrospective validation and clinical implementation of automated contouring of organs at risk in the head and neck: a step toward automated radiation treatment planning for low‐ and middle‐income countries. J Glob Oncol. 2018;4:1–11. 10.1200/JGO.18.00055 PMC6223488 30110221

